# CotA, a Multicopper Oxidase from *Bacillus pumilus* WH4, Exhibits Manganese-Oxidase Activity

**DOI:** 10.1371/journal.pone.0060573

**Published:** 2013-04-05

**Authors:** Jianmei Su, Peng Bao, Tenglong Bai, Lin Deng, Hui Wu, Fan Liu, Jin He

**Affiliations:** 1 State Key Laboratory of Agricultural Microbiology, College of Life Science and Technology, Huazhong Agricultural University, Wuhan, Hubei, People’s Republic of China; 2 Key Laboratory of Arable Land Conservation, Ministry of Agriculture, College of Resources and Environment, Huazhong Agricultural University, Wuhan, Hubei, People’s Republic of China; Instituto de Tecnologia Quimica e Biologica, Portugal

## Abstract

Multicopper oxidases (MCOs) are a family of enzymes that use copper ions as cofactors to oxidize various substrates. Previous research has demonstrated that several MCOs such as MnxG, MofA and MoxA can act as putative Mn(II) oxidases. Meanwhile, the endospore coat protein CotA from *Bacillus* species has been confirmed as a typical MCO. To study the relationship between CotA and the Mn(II) oxidation, the *cotA* gene from a highly active Mn(II)-oxidizing strain *Bacillus pumilus* WH4 was cloned and overexpressed in *Escherichia coli* strain M15. The purified CotA contained approximately four copper atoms per molecule and showed spectroscopic properties typical of blue copper oxidases. Importantly, apart from the laccase activities, the CotA also displayed substantial Mn(II)-oxidase activities both in liquid culture system and native polyacrylamide gel electrophoresis. The optimum Mn(II) oxidase activity was obtained at 53°C in HEPES buffer (pH 8.0) supplemented with 0.8 mM CuCl_2_. Besides, the addition of *o*-phenanthroline and EDTA both led to a complete suppression of Mn(II)-oxidizing activity. The specific activity of purified CotA towards Mn(II) was 0.27 U/mg. The K_m_, V_max_ and k_cat_ values towards Mn(II) were 14.85±1.17 mM, 3.01×10^−6^±0.21 M·min^−1^ and 0.32±0.02 s^−1^, respectively. Moreover, the Mn(II)-oxidizing activity of the recombinant *E. coli* strain M15-pQE-*cotA* was significantly increased when cultured both in Mn-containing K liquid medium and on agar plates. After 7-day liquid cultivation, M15-pQE-*cotA* resulted in 18.2% removal of Mn(II) from the medium. Furthermore, the biogenic Mn oxides were clearly observed on the cell surfaces of M15-pQE-*cotA* by scanning electron microscopy. To our knowledge, this is the first report that provides the direct observation of Mn(II) oxidation with the heterologously expressed protein CotA, Therefore, this novel finding not only establishes the foundation for in-depth study of Mn(II) oxidation mechanisms, but also offers a potential biocatalyst for Mn(II) removal.

## Introduction

Manganese is the second most abundant transition in the Earth’s crust and the fifth most abundant element on the Earth’s surface. Generally, manganese has three environmentally relevant oxidation states, Mn(II), Mn(III) and Mn(IV) [1], [2]. Among these, Mn(II) is thermodynamically favored at low pH and Eh, whereas Mn(III) and Mn(IV) oxides are stable at high pH and Eh [3]. The soluble form of Mn(II), serves as a crucial micronutrient for organisms, while the insoluble form of Mn(III/IV) oxide, is a highly reactive mineral phase that participates in a wide range of redox and adsorptive reactions, playing a significant role in the bioavailability and geochemical cycling of many essential or toxic elements [1]. In many environments, the chemical oxidation of Mn(II) by O_2_ in the pH range of 6.0–8.5 is at a considerably low level while in the presence of Mn(II)-oxidizing microorganisms, including a variety of bacteria and fungi, the oxidation rate can be accelerated by as much as five orders of magnitude [4], [5]. Therefore, microbial processes are considered to be primarily responsible for the formation of Mn oxides [1], [6]. Although many microorganisms capable of oxidizing Mn(II) have been isolated and belong to diverse phyla, the biochemical mechanism of Mn(II) oxidation is still enigmatic [3].

As new insights are gained regarding some proteins involved in Mn(II) oxidation, several enzymes have been gradually identified from some species of bacteria and most of them belong to a family of multicopper oxidases (MCOs). MCOs are a class of copper proteins that utilize copper as a cofactor to catalyze four one-electron oxidations of various substrates concomitantly with the reduction of O_2_ to water [7], [8]. MCOs have been found in a wide range of organisms including bacteria, fungi (laccase), plants, insects and vertebrates (ceruloplasmin) [7], [9]. So far, three model bacteria, *Bacillus* sp. strain SG-1 [6], [10], [11], *Leptothrix discophora* SS-1 [12] and *Pedomicrobium* sp. ACM3067 [13], [14], have been demonstrated to require the MCOs in Mn(II) oxidation by disruption of the corresponding genes (*mnxG*, *mofA* and *moxA*, respectively). The three MCOs identified as the putative Mn(II) oxidases show little similarity to one another outside their copper-binding motifs. In addition, the newest studies on the Mn(II)-oxidizing *alpha-proteobacterium Aurantimonas manganoxydans* SI85-9A1 and *Erythrobacter* sp. strain SD21 [4], [15] have uncovered a second class of enzymes involved in Mn(II) oxidation: heme-binding peroxidase named MopA. Another putative MCO (CumA) proposed in *Pseudomonas putida* GB-1 [16], [17], however, has been proven not to be a Mn(II) oxidase by in-frame deletion of *cumA*, and instead reveals a complex two-component regulatory pathway essential for Mn(II) oxidation in *P. putida* GB-1 [18].

To date, no bacterial Mn(II) oxidase has been purified to a large quantity sufficient for detailed biochemical study. In addition, no MCO gene thought to encode a Mn(II) oxidase has been successfully expressed in a heterologous host to yield an active enzyme [1], [5], [19], let alone the enzymological properties of the Mn(II) oxidase. To conquer these unsettled problems, our particular emphasis is placed on overexpression, purification and biochemical characterization of plentiful recombinant MCOs in *Escherichia coli*. Since most Mn(II)-oxidizing bacteria we have previously isolated belong to *Bacillus* species, which naturally strengthen our focus on the CotA (endospore coat protein A), a previously reported MCO from *B. subtilis* [20], *B. pumilus* ATCC 7061 [21] and *B. licheniformis* ATCC 14580 [22]. CotA was a classical bacterial laccase, which was able to oxidize 2,2′-azino-bis (3-ethylbenzthiazoline-6-sulphonic acid) (ABTS), syringaldazine (SGZ) and 2,6-dimethoxyphenol (2,6-DMP) [20]–[22]. To test whether the CotA is responsible for Mn(II) oxidation from a highly active Mn(II)-oxidizing strain *B. pumilus* WH4 isolated by our collaborators [23], [24], the *cotA* gene was cloned and the N-terminal His-tagged CotA was overproduced and purified. Its Mn(II) oxidase activity and enzymological properties provided the most direct evidence between the MCO and the Mn(II) oxidation. Furthermore, the Mn(II) oxidizing activities by the recombinant *E. coli* strain cultured both in Mn-containing K liquid medium system and on agar plates were also investigated comprehensively.

## Materials and Methods

### Materials

Taq DNA polymerase, restriction endonucleases and other modifying enzymes were obtained from Takara Biotechnology Co., Ltd. (Dalian, China) and Fermentas (St. Leon-Rot, Germany). Ampicillin, kanamycin, isopropyl-β-D-thiogalactoside (IPTG) and N-2-hydroxyethylpiperazine-N-2-ethanesulfonic acid (HEPES) were from Amresco (Solon, Ohio, USA). Leucoberbelin blue, ABTS, SGZ and 2,6-DMP were purchased from Sigma-Aldrich (St. Louis, MO, USA). The Coomassie (Bradford) protein assay kit was obtained from Nanjing Jiancheng Bioengineering Institute (Nanjing, China). All other chemicals were of analytical grade.

### Bacterial Strains and Culture Conditions

*B. pumilus* WH4 was isolated from Fe-Mn nodules which were collected at 40 cm depth from the subsoil horizon of a subacid orthic agrudalf developed from quaternary siliceous and alluvial sediments of the Hubei Province, Central China [23], [24]. The strain was deposited in China General Microbiological Culture Collection (CGMCC No. 2089). It was grown at 28°C in modified K medium [25], [26] made with artificial seawater [2], and supplemented with a mixture of sterile filtered 5 mM MnCl_2_ and 10 mM HEPES (pH 7.5). Control flasks without MnCl_2_ were cultured simultaneously. *E. coli* strain M15 (Qiagen, Hilden, Germany) was grown on Luria-Bertani (LB) medium at 37°C containing antibiotics as needed. Antibiotics were added to the following concentrations (µg ml^−1^): ampicillin, 100; kanamycin, 50.

### Construction of a CotA-overexpressing Strain

The *cotA* gene (accession no. JQ797387) from *B. pumilus* WH4 was amplified by PCR with the primers 5′-TTA*GGATCC*ATGAACCTAGAAAAATTTGTTGACG-3′ (forward) and 5′-CCC*AAGCTT*CTAAATAATATCCATCGGCCGCAT-3′ (reverse), the recognition sites for *Bam*HI and *Hin*dIII endonucleases are indicated in italics. The purified PCR product was digested with *Bam*HI and *Hin*dIII, and subsequently inserted into the expression vector pQE-30 (Qiagen, Hilden, Germany) that had been previously digested with the same enzymes. Consecutively, the recombinant plasmid pQE-*cotA* was transformed into *E. coli* strain M15 (Qiagen, Hilden, Germany) to obtain the recombinant strain M15-pQE-*cotA*.

### Purification of Recombinant Protein CotA

The overexpressing strain M15-pQE-*cotA* was cultivated overnight and then inoculated into LB medium containing ampicillin and kanamycin at 37°C and 180 rpm. When the optical density at 600 nm (OD_600_) reached approximately 0.6, the cells were induced with 0.5 mM IPTG and supplemented with 0.25 mM CuCl_2_. The temperature was changed to 25°C and agitation was maintained for 20 h [27]. Afterwards, cells were harvested by centrifugation (8,000 × g, 15 min, 4°C) and pellets were suspended in binding buffer (20 mM Tris-HCl, 500 mM NaCl, and 20 mM imidazole (pH 8.0)) containing 1 mg ml^−1^ lysozyme. Cells were disrupted through a French pressure cell (Aminco, Silver Spring, Maryland, USA) at 15,000 psi, followed by centrifugation (12,000×g, 30 min, 4°C) to remove cell debris. The resulting soluble extract was filtered and loaded onto nickel-chelating nitrilotriacetic acid (Ni-NTA) agarose column (Qiagen, Chatsworth, CA). The N-terminal His-tagged recombinant protein was purified following the general protocol of Ni-NTA column. The eluted fractions containing CotA were pooled, concentrated by ultrafiltration (molecular weight cutoff of 10 kDa) and analyzed by SDS-PAGE.

### Copper Content and Spectroscopic Properties

The protein concentration was determined based on the instructions of a Coomassie (Bradford) protein assay kit (Jiancheng Bioengineering Institute, Nanjing, China) using 0.563 g/l bovine serum albumin as the standard. The total copper content of purified CotA was determined by an Optima 8000 ICP-OES (inductively coupled plasma optical emission spectrometry, PerkinElmer, Norwalk, CT, USA) [28], [29]. The UV-visible absorption spectrum of purified CotA (∼33 µM) was recorded in the range of 300–800 nm in 20 mM Tris-HCl buffer (pH 8.0) at room temperature on a DU 800 UV-Vis spectrophotometer (Beckman Coulter, Inc.,Fullerton, CA, USA) [22], [30].

### Determination of Laccase Activities

To assess the laccase activity of CotA, three canonical laccase substrates ABTS, SGZ (dissolved in anhydrous ethanol) and 2,6-DMP (dissolved in 10% ethanol) were measured in 100 mM citrate-phosphate buffer (pH 4.0), 100 mM phosphate buffer (pH 6.0) and 100 mM citrate-phosphate buffer (pH 5.0), respectively [31]. Oxidation of 0.5 mM ABTS was monitored at 420 nm (ε = 36,000 M^−1^cm^−1^), of 0.6 mM SGZ at 525 nm (ε = 65,000 M^−1^cm^−1^) and of 2 mM 2,6-DMP at 468 nm (ε = 49,600 M^−1^cm^−1^) [32]. Since CotA was evolved to accept ABTS over SGZ [33], the following experiments were carried out with ABTS to characterize the enzyme activities in detail. Unless otherwise indicated, the standard reaction mixture (1 ml) contained 100 mM citrate-phosphate buffer (pH 4.0), 0.5 mM ABTS and 93 µg purified recombinant CotA. The reaction mixtures were incubated at 37°C and 180 rpm for 5 min in a shaking incubator. Thereafter, the reaction mixture was measured at 420 nm [34]. Enzyme activity measurements were performed either on a DU 800 UV/Vis spectrophotometer (Beckman Coulter, Inc., USA) or on a Multiskan spectrum microplate reader (Thermo Fisher Scientific Inc., Waltham, MA, USA) with a 96-well plate. All assays were performed in triplicate. One unit of laccase activity was defined as the amount of enzyme that produced 1 µmol of product per minute under the standard assay conditions [21], [22].

The effect of pH on enzyme activity was determined at 37°C in 100 mM citrate-phosphate buffer (pH 3.0–8.0) for the ABTS. The temperature optimum for the activity was performed at temperatures ranging from 30 to 100°C by measuring ABTS oxidation. The copper requirement was tested by adding CuCl_2_ (0–3 mM) to the standard reaction mixtures [34].

Kinetic parameters for the purified recombinant CotA were determined at room temperature by using different concentrations of ABTS (20–460 µM) [35]. The initial rates, recorded within 3 to 10 min, were acquired from the linear portion of the reaction curve. The kinetic parameters were calculated by non-linear regression fitting of the data to the Michaelis–Menten equation using the software Graphpad Prism 5 (GraphPad Software, San Diego, CA).

### Mn(II) Oxidase Assays in Liquid Culture System

Mn(II) oxidation assays in liquid culture system were routinely performed as follows: 1 ml of initial reaction mixture (10 mM HEPES (pH 8.0) containing 5 mM MnCl_2_ and 0.093 mg ml^−1^ purified recombinant CotA) was incubated at 37°C for about 24 h. Mn(II) oxidation was monitored by the formation of brown or black Mn oxides, and then checked by Leucoberbelin blue reagent which was an acidified redox dye and could be specifically oxidized by Mn with valences of +3 or higher, resulting in a blue color product with an absorption maximum at 620 nm [36]. For the quantification of the biogenic Mn oxides, 50 µl of each sample was reacted with 250 µl of Leucoberbelin blue solution, and then the Mn (IV) oxide concentration was calculated by multiplying with a factor of 2.5 according to the KMnO_4_ concentration in standard curve. The measurement was performed either on a DU 800 UV-Vis spectrophotometer (Beckman Coulter, Inc., USA) or on a Multiskan Spectrum spectrophotometer (Thermo Scientific, Vantaa, Finland) with a 96-well plate. All assays were performed in triplicate.

The optimum pH value for Mn(II) oxidizing activity of CotA was determined by varying the pH of reaction buffers. In this assay, two different reaction buffers 10 mM HEPES and 100 mM CHES, were used for pH ranges of 6.8–8.2 and 8.6–9.5, respectively. Other reaction conditions were the same as described above. To study the effect of temperature, the assay was carried out at temperatures ranging from 28 to 85°C. The optimization of Cu^2+^ concentration was evaluated by adding CuCl_2_ (0.05–1.4 mM) to the initial reaction mixtures. All assays were performed in triplicate.

To investigate whether the Mn(II)-oxidizing activity of CotA was solely depended on copper, each of 1 mM divalent metal cations (Sr^2+^, Zn^2+^, Ca^2+^, Ba^2+^, Mg^2+^, Ni^2+^ and Hg^2+^) was separately tested by the replacement of 1 mM Cu^2+^. After individually pre-treating them with purified CotA for 1 h at 37°C, the metal-enzyme complexes were added into 10 mM HEPES (pH 8.0) containing 5 mM MnCl_2_ at 37°C for 24 h and the residual enzyme activities were measured, respectively. The enzymatic activity assayed with CuCl_2_ was taken to be 100%. All assays were performed in triplicate.

The effect of various organic compounds (1 mM EDTA, 1 mM DTT, 1 mM guanidine-HCl, 1% SDS or 0.1 mM *o*-phenanthroline) on the enzyme activity was also studied. It was performed by pre-incubating the enzyme with each reagent at 37°C for 1 h without substrate, then added to 10 mM HEPES (pH 8.0) containing 5 mM MnCl_2_ and 0.8 mM CuCl_2_ to measure the remaining Mn(II)-oxidizing activity. The activity assayed in the absence of the reagent was taken to be 100%. All assays were performed in triplicate.

### Kinetic Parameters of Mn(II) Oxidizing Activity

The kinetic parameters were determined by measuring the initial reaction velocity at various concentrations of MnCl_2_ ranging from 2.5 to 50 mM in 10 mM HEPES (pH 8.0) containing 0.8 mM CuCl_2_ at 37°C. For each Mn(II) concentration, we measured the production of Mn oxides at regular intervals and then drew the curve. In order to get the accurate initial rate (V_0_) of enzymatic reaction, the reaction time was chosen before 5% of the Mn(II) was oxidized. The initial rates (V_0_) of different Mn(II) concentrations was obtained from the slope of the Mn oxide process curve. The K_m_ and V_max_ were calculated with GraphPad Prism 5 (GraphPad Software, San Diego, CA), using standard settings for non-linear regression curve fitting in Michaelis-Menten modus. The k_cat_ parameter was determined using the equation k_cat_ = V_max_/[E] ([E] = 1.55×10^−7 ^M). One unit of Mn(II)-oxidizing activity was defined as the amount of enzyme that produced 1 µmol of product per minute under the standard assay conditions. All assays were performed in triplicate.

### Mn(II) Oxidation in-gel Activity Assay

The purified recombinant protein was subjected to native polyacrylamide gel electrophoresis (PAGE) using 12% polyacrylamide gels and Tris glycine buffer [9], [37]. Native PAGE was conducted with two sets of samples running side by side without β-mercaptoethanol, SDS, and sample boiling. After electrophoresis, the gel was sliced into two pieces. One half was stained with Coomassie blue and the other half was assayed for Mn(II) oxidase activity. For measuring the Mn(II) oxidation, the gel was first immersed in prewash solution containing 10% glycerol and 0.5% Triton X-100 for 30 min, and then replaced with 10 mM HEPES buffer (pH 7.5) to incubate the gel at room temperature for 10 min [15]. Finally, the gel was incubated overnight in 10 mM HEPES buffer (pH 7.5) with 5 mM MnCl_2_ and 0.8 mM CuCl_2_ at room temprature. The Mn(II) oxidase activity could be determined by forming a visible brown band, which was at the same position of Coomassie blue-stained gel.

### Mn(II) Oxidation Assay of the Recombinant *E. coli* Strain

The mother strain M15 and recombinant strain M15-pQE-*cotA* were separately grown overnight at 37°C in K medium containing corresponding antibiotics. A portion of the culture was then inoculated into fresh K medium (1∶100 dilution) with 10 mM HEPES (pH 7.5) and antibiotics if needed. The Mn(II) oxidation assays were performed in the presence of 5 mM MnCl_2_ and the reaction media without MnCl_2_ were set as a negative control. After growing to an OD_600_ of 0.6–0.8, IPTG and CuCl_2_ were added to the final concentrations of 0.5 mM and 0.25 mM, respectively. The cultures were then shaken at 28°C for 7 days and 100 µl aliquots were taken and measured every day: the cells were harvested by centrifugation at 12,000×g for 5 min and were resuspended in 10 mM HEPES (pH 7.5). The suspensions were reacted with Leucoberbelin blue for 10 min and the absorbances of the supernatants were measured at 620 nm [36]. The biogenic Mn oxides were calculated by the KMnO_4_ standard curve. Besides, the supernatant of 7-day cultivated culture was also measured by an ICP-OES (PerkinElmer, Norwalk, CT, USA) to assess the residual Mn(II). Another aliquots (5 µl) of the overnight cultures were then spotted onto K plates supplemented with 5 mM MnCl_2_ and 0.5 mM IPTG. After incubation at 28°C for 7 days, the plates were photographed before and after the addition of Leucoberbelin blue to record the extent of Mn(II) oxidation. All assays were performed in triplicate.

### Scanning Electron Microscopy (SEM)

The *E. coli* strains M15 and M15-pQE-*cotA* cultivated with and without Mn(II) were collected by centrifugation after 7 days of cultivation and pretreated before SEM. The SEM samples were washed with phosphate buffer (pH 7.2) for 3 times, and then fixed with 2.5% (v/v) glutaraldehyde overnight at 4°C, followed by dehydration with an ethanol series (30%, 50%, 70%, 80%, 90%, and 100%; every step was performed twice and continued for 15 min). Subsequently, the samples were freeze-dried at −100°C for 24 h and stored in a desiccator before measurement. Cell morphologies and the biogenic Mn oxides produced on the cell surface, were examined under a JSM-6390/LV scanning electron microscope (SEM; JEOL, Japan) with 20,000 V accelerating voltage [38].

## Results

### The Sequence Characteristics of CotA

Until now, there was no complete and annotated genome sequence available for *B. pumilus* WH4 strain. By searching the nucleotide and protein databases, we have identfied a CotA (YP_001485796.1) sequence in the published genome sequence of *B. pumilus* SAFR-032. The identity in amino acid sequence is 98% with the *B. pumilus* ATCC 7061 (ZP_03054403.1) [21], which was recently demonstrated to be a laccase-like MCO with similar properties as CotA of *B. subtilis* [20]. 16 S rDNA sequences alignment and phylogenetic tree analysis showed that the *B. pumilus* WH4 strain was closely (>97%) related to *B. pumilus* SAFR-032. So the primers were designed according to the most similar *cotA* sequence of the *B. pumilus* SAFR-032. The *cotA* gene was amplified by PCR using genomic DNA from *B. pumilus* WH4 as template and 1530 bp PCR product was obtained. Subsequently, the gene was inserted into vector pQE-30 and transformed to host strain *E. coli* M15.

**Figure 1:**
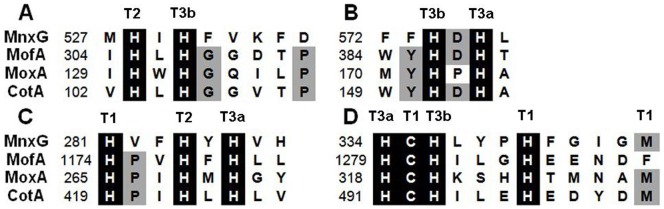
Amino acid sequence alignment of the four conserved copper-binding sites of MCOs from diverse strains. The copper-binding regions A, B, C and D correspond to those shown in Figure S1. The copper-binding residues are designated T1, T2, T3a and T3b on the basis of the types of copper which they potentially bind. MnxG, marine *Bacillus* sp. strain SG-1 MnxG (GenBank accession no. AAB06489.1); MofA, *L. discophora* SS-1 MofA (GenBank accession no. CAA81037.2); MoxA, *Pedomicrobium* sp. ACM 3067 MoxA (GenBank accession no. CAJ19378.1); CotA, *B. pumilus* WH4 CotA (this study, GenBank accession no. AFL56752.1). Conserved amino acids are shaded in black (90% conservation or more) or in grey (70 to 90% conservation).

Amino acid sequence analysis revealed that the CotA protein contained four conserved copper-binding motifs (Figures 1 and S1). Moreover, the copper-binding motifs of CotA shared a significant identity with other MCOs, but the similarity of the remainder of the proteins was quite poor (Figures 1 and S1).

On the other hand, CotA also shows some similarities as well as differences in comparison with the well investigated Mn(II) oxidases such as MnxG, MofA and MoxA (Figure S1). First of all, CotA (509 aa) is much shorter than MnxG and MofA (1217 aa and 1661 aa, respectively) which are both large proteins containing over one thousand amino acids [11], [12], but it is similar with MoxA (476 aa) [14]. Moreover, the copper-binding residues are usually located at the N- and C-terminus for many MCOs. In this gene, the four conserved copper-binding motifs, A, B, C and D, are transcribed in a same order as MofA and MoxA: A and B motifs are existed at the N-terminus, C and D motifs are present at the C-terminus. However, this order feature is transcribed in reverse in MnxG of *Bacillus* sp. SG-1 and related strains, where A and B motifs are located at the C-terminus while C and D motifs are located at the N-terminus [11]. Furthermore, there is also no copper-binding F motif in CotA, which is predicted to be the fifth copper-binding motif other than the four copper-binding motifs, even though the function of F motif is still unknown [6].

### Purification and Biochemical Properties

**Figure 2:**
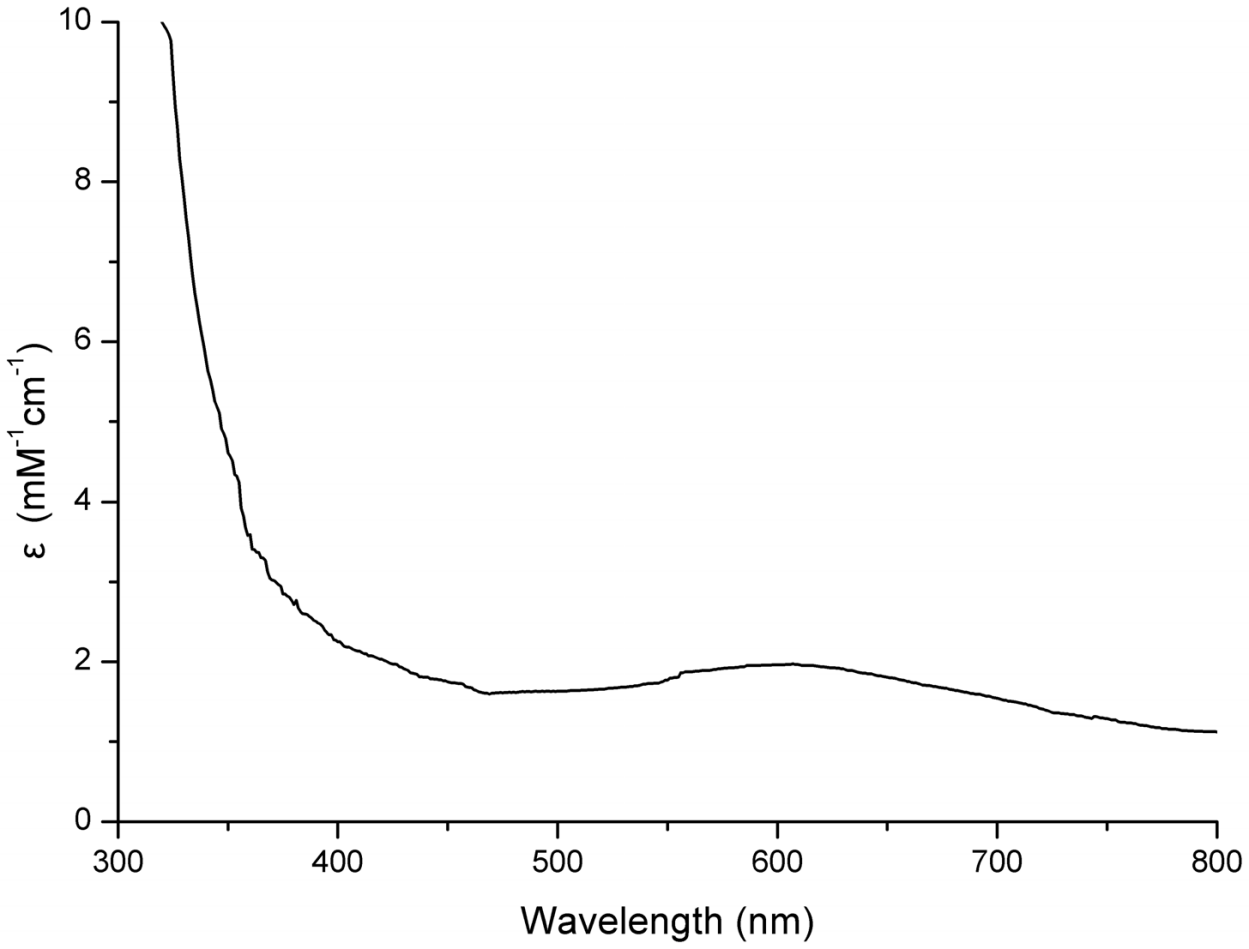
The UV-visible spectrum of the purified recombinant CotA. Optical absorbance spectroscopy of purified CotA was recorded on a DU 800 UV-Vis spectrophotometer. Pure CotA (∼33 µM) in 20 mM Tris-HCl buffer (pH 8.0) was scanned in 1 cm quartz cuvettes at room temperature.

The overexpressing strain M15-pQE-*cotA* was induced with 0.5 mM IPTG at 25°C with the addition of 0.25 mM Cu^2+^, which provided an appropriate state for folding and yielding a fully copper incorporated holoenzyme [27]. After purification, the concentration of the recombinant CotA can reach 0.78 mg ml^−1^, and a single protein band with a molecular weight of 60 kDa was detected by SDS-PAGE (Figure S2). The UV-visible spectrum of the purified CotA showed the traditional band at 607 nm (Figure 2), and the ratio of copper atoms/molecule of CotA was calculated to be 3.49±0.05 by ICP-OES, indicating that it was a typical blue copper oxidases.

### The Laccase Activity of CotA

We next investigated the laccase activity by oxidizing three classical substrates ABTS, SGZ and 2,6-DMP, respectively. Like the other MCOs [20], [28], [31], CotA was able to oxidize these specific substrates, indicating a robust laccase activity (Figure S3). It was supported likewise by the fact that CotA appeared to have a flexible lidlike region close to the substrate-binding site that may mediate substrate accessibility [39]. The maximum oxidizing activity towards ABTS was observed at 73°C in 100 mM citrate-phosphate buffer (pH 4.0) containing 1 mM CuCl_2_ (Figure S4), it was approximately consistent with the studies in *B. subtilis*, *B. pumilus* and *B. licheniformis* [21], [22], [34], suggesting that CotA was a thermoactive and copper-dependent enzyme. The highest specific activity of the CotA ([E] = 1.30×10^−7 ^M) with the ABTS substrate was 0.15 U mg^−1^. Kinetic constants K_m_, V_max_ and k_cat_ for ABTS were 35.24±3.17 µM, 1.22×10^−6^±0.05 M·min^−1^ and 0.16±0.01 s^−1^, the K_m_ value was very similar to the previous observations, while k_cat_ value was much smaller then CotA [21], [32], [34] from other *Bacillus* sp. strains. A possible explanation for these differences was that the experiments were performed in different enzymes and reaction systems.

### Mn(II) Oxidase Activities Determined in Liquid Culture System *in vitro*

**Figure 3:**
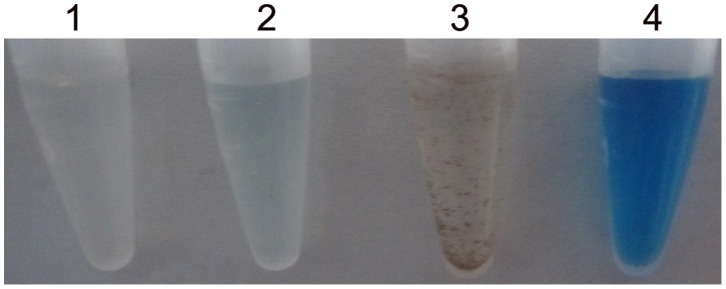
Mn(II) oxidase activity of purified recombinant CotA in liquid culture system. Tube 1, 10 mM HEPES (pH 8.0) plus 5 mM MnCl_2_ and 0.8 mM CuCl_2_ (reaction mixture); tube 2, aliquots (50 µl) of tube 1 reacting with 250 µl Leucoberbelin blue; tube 3, reaction mixture in Tube 1 plus CotA; tube 4, aliquots (50 µl) of tube 3 reacting with 250 µl Leucoberbelin blue.

Mn(II) oxidation by purified recombinant CotA in liquid culture system *in vitro* was firstly established in this study. When CotA was incubated at 37°C in optimum reaction mixtures for about 24 h, it was easy to find that lots of brown or black precipitates were formed in the solution after exposure to Mn(II), and these precipitates were confirmed to be Mn oxides by the Leucoberbelin blue assay (Figure 3).

**Figure 4:**
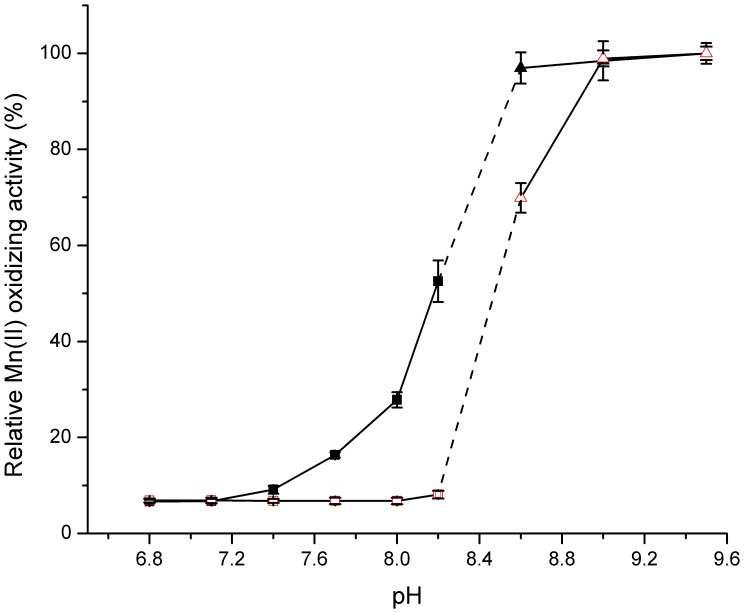
Effect of pH on Mn(II)-oxidizing activity of CotA. ▪ (black square) CotA plus 5 mM MnCl_2_ and 0.8 mM CuCl_2_ in HEPES buffer (pH 6.8–8.2) at 37°C for about 24 h; □ (red square) control test with 5 mM MnCl_2_ and 0.8 mM CuCl_2_ in HEPES buffer (pH 6.8–8.2) at 37°C for about 24 h; ▴ (black triangle) CotA plus 5 mM MnCl_2_ and 0.8 mM CuCl_2_ in CHES buffer (pH 8.6–9.5) at 37°C for about 24 h; △ (red triangle) control test with 5 mM MnCl_2_ and 0.8 mM CuCl_2_ in CHES buffer (pH 8.6–9.5) at 37°C for about 24 h. The values were means ± standard deviations for triplicate assays.

**Figure 5:**
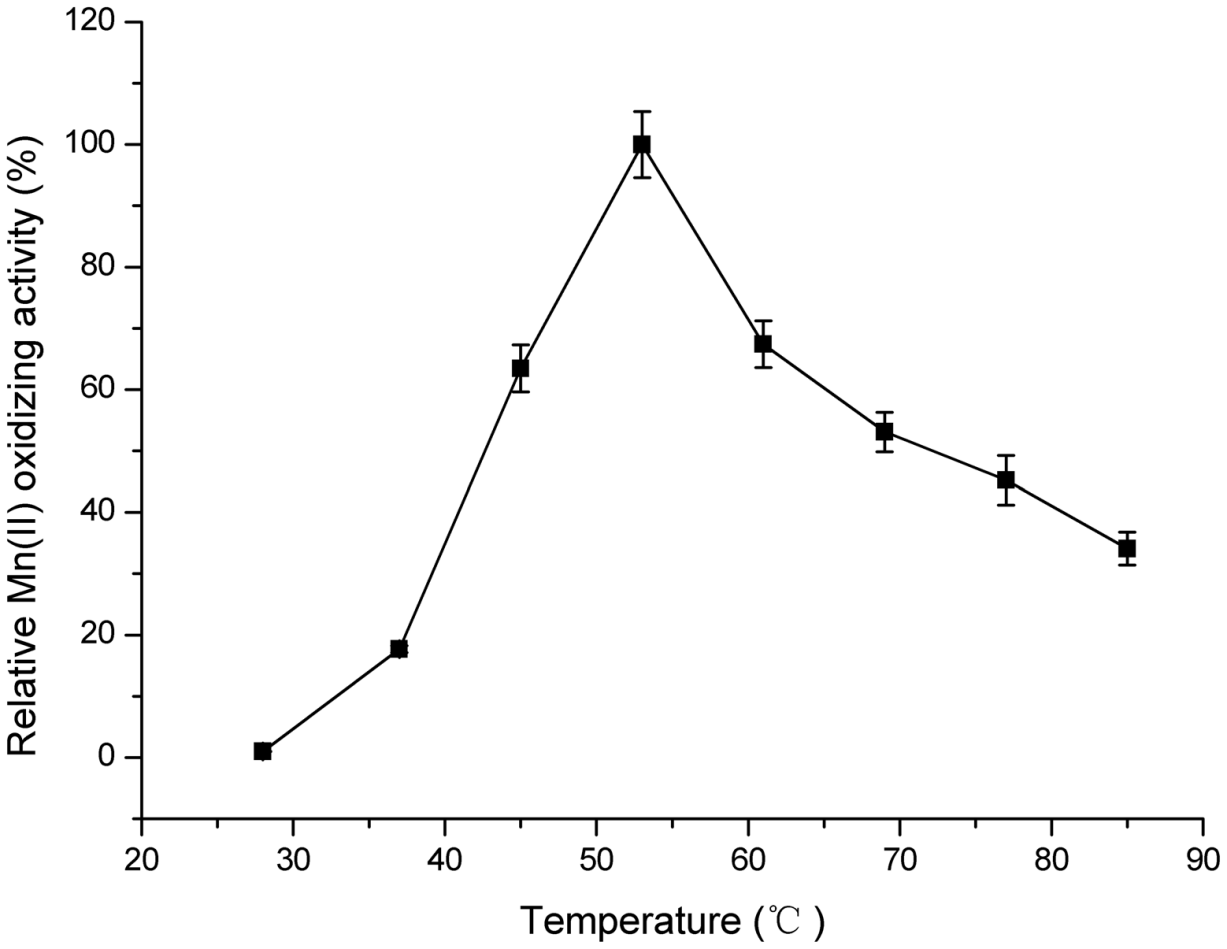
Effect of temperature on Mn(II)-oxidizing activity of CotA. Enzyme reactions were carried out in HEPES buffer (pH 8.0) supplemented with 5 mM MnCl_2_ and 0.8 mM CuCl_2_ at temperatures ranging from 28 to 85°C. The values were means ± standard deviations for triplicate assays.

**Figure 6:**
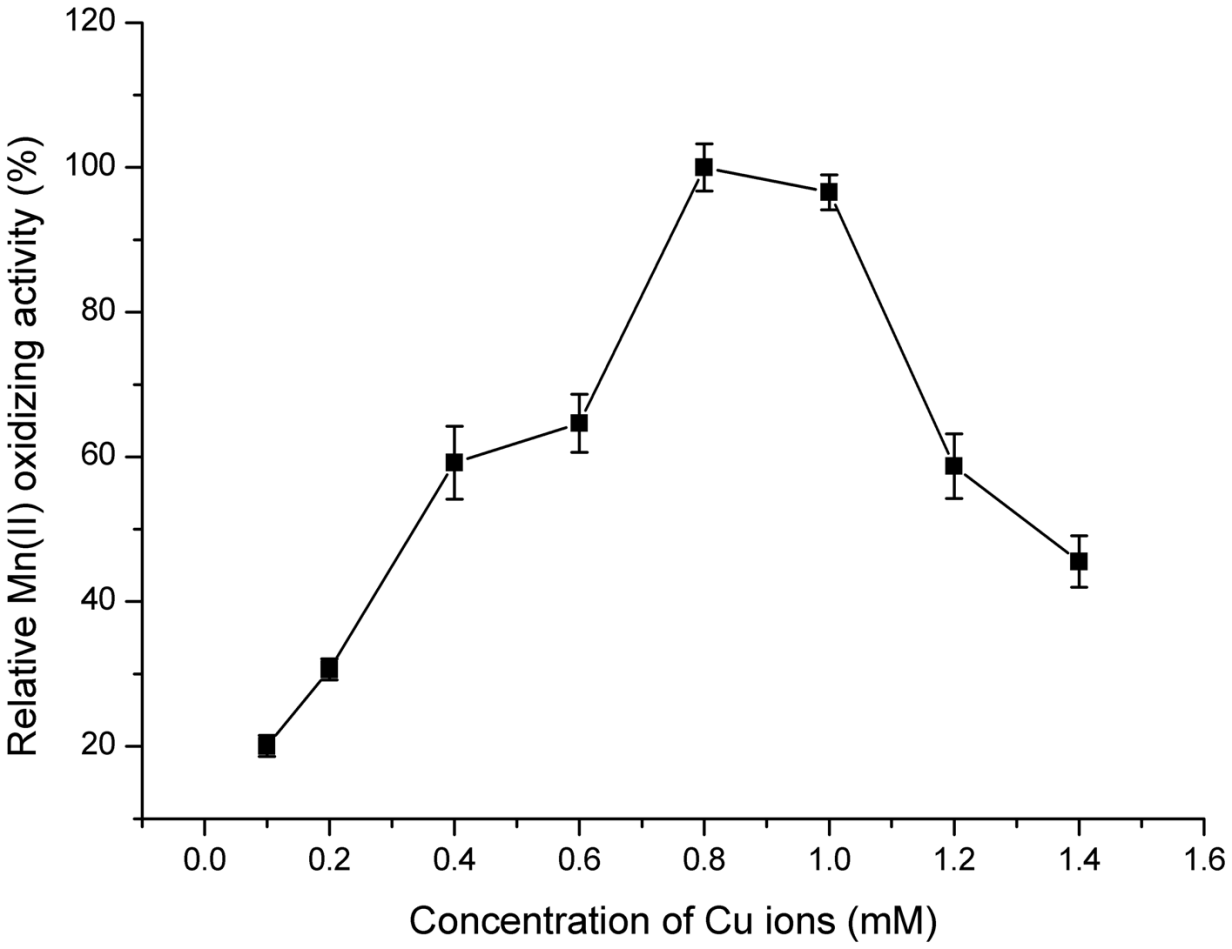
Effect of copper concentration on Mn(II) oxidation of CotA. Enzyme reactions were performed in HEPES buffer (pH 8.0) supplemented with 5 mM MnCl_2_ and various copper concentrations (0.05–1.4 mM). The values were means ± standard deviations for triplicate assays.

It can be seen from Figure 4 that the enzyme activity was strongly affected by pH. The *optimum pH* range for Mn(II)-oxidizing activity was *from* 7.5 to 8.0 without any autoxidation in the control sample, while few Mn oxides were detected below pH 7.0. This pH value was very similar to those observed in *P. putida* GB-1 [40], *Pedomicrobium* sp. ACM 3067 [13], *Bacillus* sp. SG-1 [26] and *L. discophora* [25], which all displayed an optimum Mn(II)-oxidizing activity at *pH* values *from* 7.0 to 8.0. Notably, when pH value *exceeded 8.5*, the Mn(II)-oxidizing activity increased simultaneously with elevated pH regardless of whether CotA was added or not. It was principally a *result of* autoxidation [13], [26] and thus *pH 8.0* was *selected* for the further study. Furthermore, the rate of enzymatic reaction was extremely slow below 28°C, and asymptotically approached *its maximum* at 53°C (Figure 5). However, CotA showed a declined stability when it was incubated at higher temperatures for 2–5 h (data not shown). To reach a compromise between activity and stability of the enzyme, 37°C was chosen as the moderate reaction temperature in the subsequent experiments. Among all the metal ions that tested for their effects on enzymatic activity (Table 1), only Cu^2+^ significantly enhanced the Mn(II) oxidizing activity of CotA, and the highest activity was obtained at 0.8 mM Cu^2+^ (Figure 6). It coincided pretty well with the previous studies that copper indeed had a profound stimulating effect on the oxidation of Mn(II) [41]. This positive role of Cu^2+^ might be interpreted by the possibility that Cu^2+^ was the essential cofactor and enhanced the folding of CotA (Figure S7) [42].

**Table 1:** Effects of metal ions on the Mn(II)-oxidizing activity of purified CotA.

Reagents	Concentration	Relative activity
CuCl_2_	1.0****mM	100%
SrCl_2_	1.0 mM	1.39±0.01%
ZnCl_2_	1.0 mM	0.46±0.02%
CaCl_2_	1.0 mM	0.83±0.04%
BaCl_2_	1.0 mM	1.17±0.08%
MgCl_2_	1.0 mM	0.85±0.03%
NiSO_4_	1.0 mM	0.76±0.02%
HgCl_2_	1.0 mM	0.77±0.03%

The values were means ± standard deviations for triplicate assays.

On the other hand, the addition of classical metal chelators such as *o*-phenanthroline and EDTA both led to a complete loss of Mn(II)-oxidizing activity (Table 2), pointing to involvement of a metal cofactor in the Mn(II) oxidation process [26], [40], [43]. The Mn(II)-oxidizing activity was also negatively affected by SDS [25] or DTT (Table 2).

**Table 2:** Effects of organic compounds on the Mn(II)-oxidizing activity of purified CotA.

Reagents	Concentration	Relative activity
CuCl_2_	1.0 mM	100%
EDTA	1.0 mM	2.36±0.10%
DTT	1.0 mM	54.75±2.51%
guanidine-HCl	1.0 mM	87.30±4.59%
SDS	1.0%	38.78±1.41%
*o*-phenanthroline	0.1 mM	2.71±0.06%

The values were means ± standard deviations for triplicate assays.

The specific activity of purified CotA towards Mn(II) was 0.27 U/mg. The K_m_, V_max_ and k_cat_ values towards Mn(II) were 14.85±1.17 mM, 3.01×10^−6^±0.21 M·min^−1^ and 0.32±0.02 s^−1^, respectively.

### In-gel Mn(II)-oxidizing Activity

**Figure 7:**
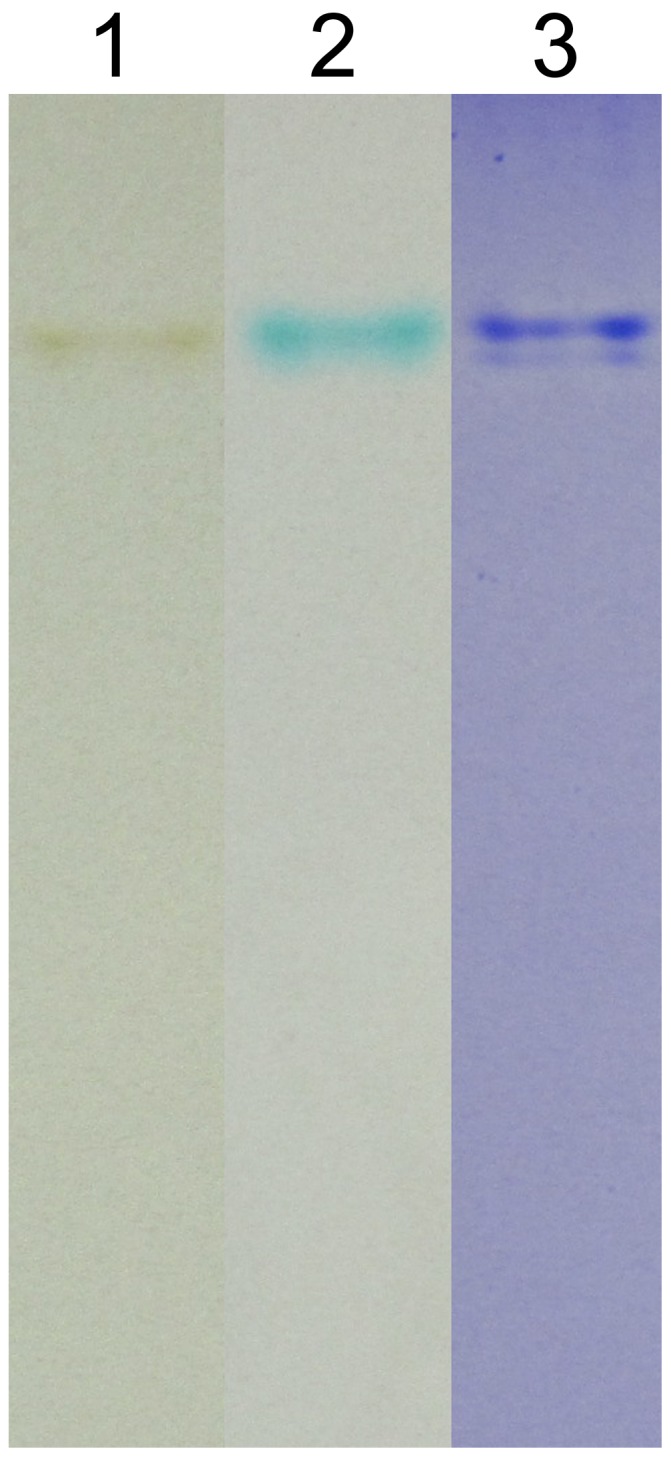
Native PAGE gel analysis of purified recombinant CotA. Lane 1, in-gel Mn oxidation assay; lane 2, in-gel Mn oxidation assay stained with Leucoberbelin blue; lane 3, coomassie blue-staining assay.

Mn(II)-oxidizing activity analysis of CotA protein using native PAGE in-gel activity assay was performed. The purified recombinant CotA yielded a brown band which agreed well with the corresponding Coomassie blue-stained band (Figure 7). Moreover, the brown band turned blue when the Leucoberbelin blue was added, conclusively indicating that Mn oxides just deposited on the enzyme band.

### Oxidation of Mn(II) by the Recombinant *E. coli* Strain

**Figure 8:**
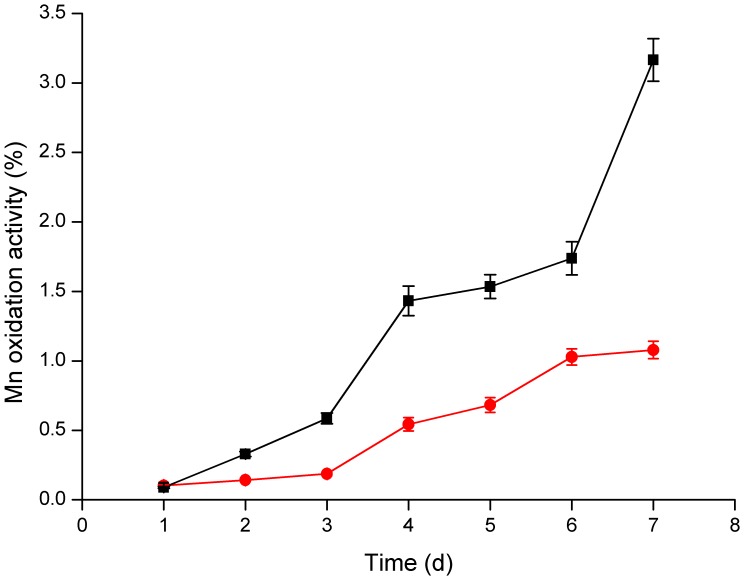
Determination of the Mn(II) oxidation activity of *E. coli* strains every day. *E. coli* strains M15-pQE-*cotA* (▪) (black square) and M15 (•) (red circle) were grown at 37°C in K liquid medium containing HEPES (pH 7.5), 0.5 mM IPTG, 0.25 mM CuCl_2_ and 5 mM MnCl_2_ for 7 days. The values were means ± standard deviations for triplicate assays.

**Figure 9:**
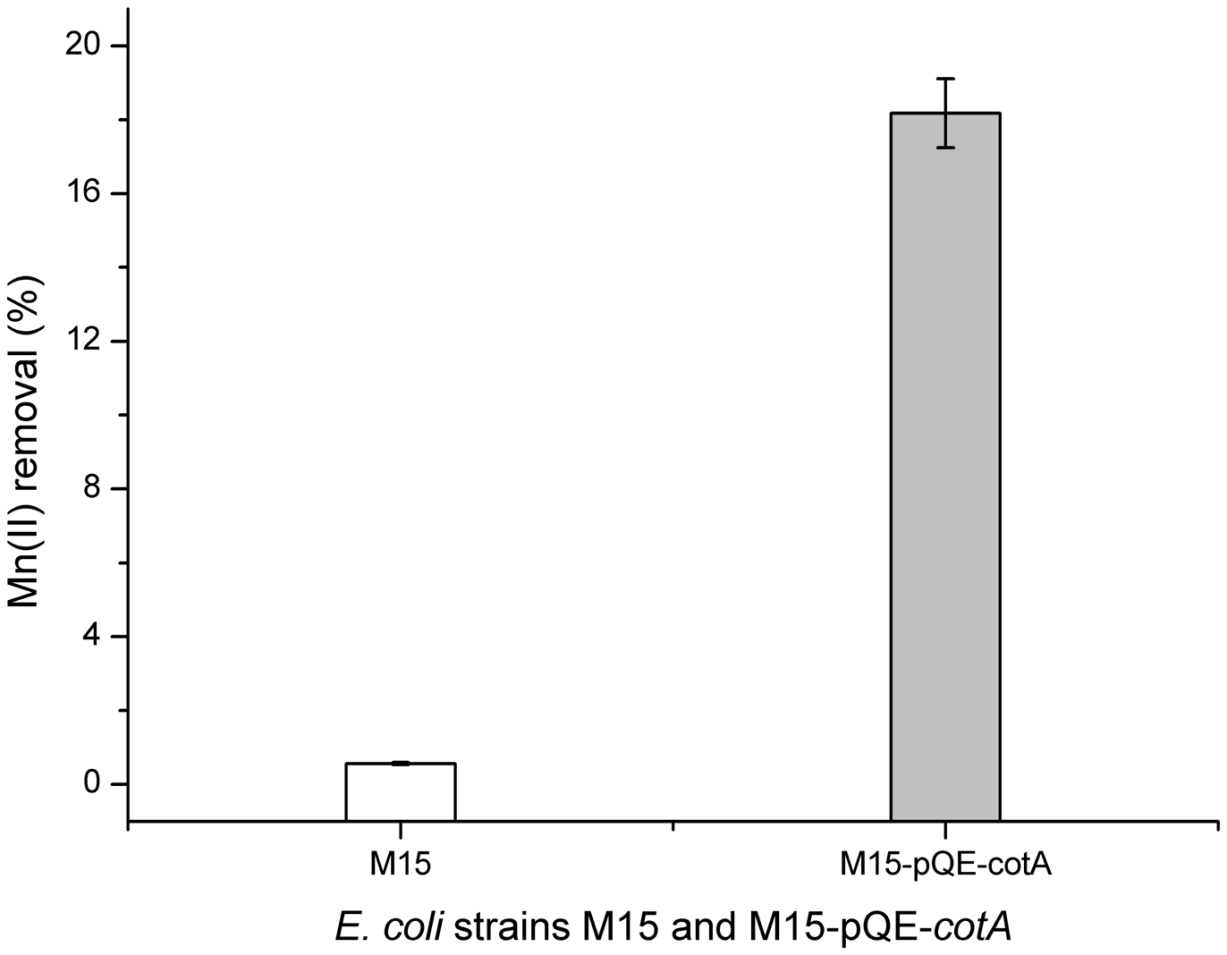
The Mn(II) removal percentages from the supernatants by *E. coli* strains M15-pQE-*cotA* and M15. The residual Mn(II) of the 7-day cultivated culture was measured by ICP-OES. The values were means ± standard deviations for triplicate assays.

On K agar plates, *E. coli* strains M15 and M15-pQE-*cotA* were grown with the addition of IPTG, which could induce the overexpression of CotA. Strikingly, after 7 days of cultivation in the presence of Mn(II), the recombinant strain M15-pQE-*cotA* showed the characteristic brown color resulting from the accumulation of Mn oxides on the bacterial surfaces (Figure S5A2) and showed apparent blue after the detection by Leucoberbelin blue (Figure S5B2). While in the absence of Mn(II), the precipitates were not observed (Figure S5A1) and did not turn blue after reaction with Leucoberbelin blue (Figure S5B1). As a control, the mother strain M15 cultivated in the presence of Mn(II) displayed nearly whitish (Figure S5C2) and only changed to a weak blue after the addition of Leucoberbelin blue (Figure S5D2). In order to further explore the Mn oxidizing ability of M15 and M15-pQE-*cotA*, both stains were grown in K liquid culture medium. After 7 days, the mother strain M15 showed a weak Mn(II) oxidizing activity and only 1.08% of Mn oxides were produced (Figure 8). While for the recombinant strain M15-pQE-*cotA*, the Mn(II) oxidizing activity was much stronger, and yielded about 3.16% of Mn oxides. Additionally, the soluble Mn(II) of the 7-day cultivated supernatant were determined accurately by ICP-OES. As illustrated in Figure 9, 18.2% dissolved Mn(II) was removed by M15-pQE-*cotA*, which appeared to be 32 times greater than that of the mother strain (0.56%).

### SEM Analysis of Cell Morphologies and Mn Oxides

**Figure 10:**
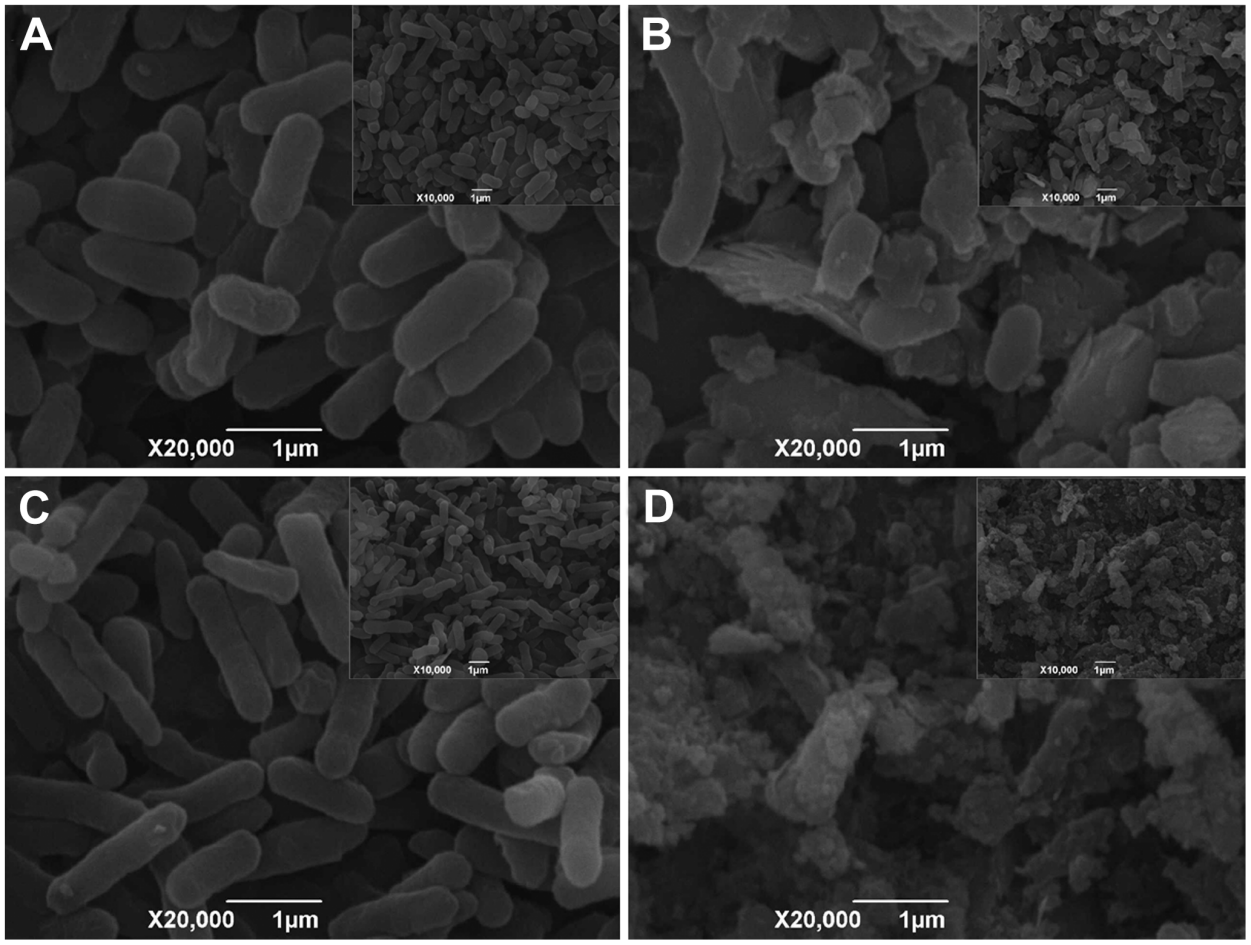
SEM photographs of *E. coli* cells and biogenic Mn oxides (×20,000 with insert ×10,000). (A) SEM image showing the morphologies of the mother strain M15 cultivated without Mn(II); (B) SEM image of the mother strain M15 cultivated with Mn(II) and the associated biogenic Mn oxides; (C) SEM image showing the morphologies of the recombinant strain M15-pQE-*cotA* cultivated without Mn(II); (D) SEM image of the recombinant strain M15-pQE-*cotA* cultivated with Mn(II) and the aggregated biogenic Mn oxides.

From the SEM photographs, we can clearly observe that both the *E. coli* strains M15 (Figure 10A) and M15-pQE-*cotA* (Figure 10C) behave in physiologically normal fashions after cultivated for 7 days without Mn(II). However, the addition of Mn(II) directly influences the cell morphology, it leads to the pronounced morphological irregularities because the Mn oxide is occurred as a precipitate covering the cell surface (Figure 10B and 10D). The SEM images show that the accumulation of biogenic Mn oxides located on recombinant strain M15-pQE-*cotA* is much more when compared with the mother strain M15. On the cell surfaces of M15, only a few precipitates can be observed, while many Mn oxides are deposited outside the cells of M15-pQE-*cotA*, and therefore the strains are heavily encrusted with Mn oxides. These biogenic Mn oxides are aggregated particles with irregular geometric shapes, indicating their poor crystallinity and very small particle size.

## Discussion

### CotA from *B. pumilus* WH4 is Firstly a Bacterial Laccase

Previous studies demonstrated that CotA was able to oxidize ABTS, SGZ and 2,6-DMP [22], [34], [44]. In this study, the recombinant CotA oxidized the classical laccase substrates similarly to the other *Bacillus* CotA enzymes.

Amino acid sequence alignment of the CotA from *B. pumilus* WH4 with other CotA protein origins shows that the copper-binding motifs are alike [33], [45]. Moreover, the CotA from *B. pumilus* WH4 (AFL56752.1) displays a very high sequence identities (96% and 97%) with the recently reported CotA from *B. pumilus* ATCC 7061 [21] and *B. pumilus* SAFR-032, respectively. It also shows 68% and 61% identities with the CotA proteins from *B. subtilis* 168 (NP_388511.1) [20] and *B. licheniformis* ATCC 14580 (YP_077905.1) [22], respectively (Figure S6). The homology model of CotA from *B. pumilus* WH4 (Figure S7A) is constructed using SWISS-MODEL program based on its homologous template from *B. subtilis* (2WSD) [39]. Three-dimensional structure models of the CotA and the template protein are superposed very well within the four copper-binding motifs. The residues containing H103, H105, H151, H153, H419, H422, H424, H491, C492, H493, H497, and M502 are involved in copper ion binding (Figure S7B). The coordination bonds among the 4 copper ions and the 12 conserved amino acid residues of CotA are shown in plane (Figure S7C). Thus, copper ions and copper-binding motifs constitute the active center and benefit the stability of the structure.

### The Purified Recombinant CotA Exhibits Manganese-oxidase Activity *in vitro*

Experimental evidences have demonstrated that bacterial Mn(II) oxidation is an enzymatic process [13], and many MCOs are postulated to be directly involved. In none of these cases, however, have the Mn(II)-oxidizing macromolecules been purified to such an extent as to allow further studying of biochemical characteristics [40]. Thus, quick and efficient methods for purifying enough Mn(II) oxidases are needed urgently and surely have been gained much attention in recent years [7]. In *L. discophora* SS-1, a small amount of Mn(II)-oxidizing protein was isolated from the polyacrylamide gel by electroelution [12]. Furthermore, a three-step purification strategy consisting of ion exchange, hydrophobic interaction, and size exclusion chromatographies, was used to separate the Mn oxidase from the loosely bound outer membrane protein fraction in both *A. manganoxydans* strain SI85-9A1 and *Erythrobacter* sp. strain SD-21 [15]. However, these methods are much more laborious and ineffective for the purification of larger amounts of enzyme compared with heterologous overexpression. To date, attempts to purify active Mn(II) oxidases by genetic manipulation in *E. coli* strain have not yet succeeded [6], [12]. Neither MnxG, MofA, nor CumA is able to oxidize Mn(II) when produced from an expression vector in *E. coli* [7]. As a result, elucidation of their roles in the biochemical mechanism of Mn oxidation awaits the breakthrough in purification of active Mn(II) oxidase [6].

In this study, we harvested enough recombinant CotA by heterologous overexpression. Subsequently, we successfully verified that CotA was directly involved in Mn(II) oxidation. Previously, the kinetic parameters for the oxidation of Mn(II) were obtained either from whole cells (spores) or cell extracts, and none of the research had been conducted with the purified recombinant enzyme. In the present study, kinetic constants K_m_, V_max_ and k_cat_ values of purified CotA towards Mn(II) were 14.85±1.17 mM, 3.01×10^−6^±0.21 M·min^−1^ and 0.32±0.02 s^−1^, respectively. The apparent K_m_ was much higher when compared with the study carried out with the whole cells from *Pedomicrobium* sp. ACM 3067 (26 µm) [13]. On the other hand, the results of K_m_ and V_max_ values were a bit similar to the previous observations made by Douka [46] who used the cell extracts of two bacterial strains to oxidize Mn(II) (3.3 mM and 20×10^−6^ M·min^−1^, respectively). While for k_cat_ value, little information is available on Mn(II) oxidation till now.

For the native PAGE in-gel activity assays, the purified recombinant CotA produced a brown Mn oxide band in line with the relevant coomassie blue-stained band. These results, combined with the asssy in liquid culture system, further confirmed that CotA had the Mn(II) oxidase activity.

Consequently, we believe that our data are the first direct observation of Mn(II) oxidation with the heterologously expressed protein *in vitro*, thereby giving a thorough understanding of the enzymological properties of CotA that relates to Mn(II) oxidation. Our result also put forward an effective overproduction system and a practical purification protocol for active Mn(II) oxidases, which will open the way for spectroscopic and eventually crystallographic characteristics of these putative MCOs and their Mn oxides [7].

### The Recombinant *E. coli* Strain has Higher Mn(II)-oxidizing Activity

To testify this crucial role *in vivo* of *cotA*, the recombinant strain M15-pQE-*cotA* was cultured both in Mn(II)-containing K liquid medium and on the K agar plates. The results clearly indicated that the colony color of M15-pQE-*cotA* was shifted from whitish to brownish [47], while the mother strain M15 remained nearly whitish. No change in colony color was observed when M15-pQE-*cotA* was grown in the absence of Mn(II). In addition, the results of Mn removal efficiency and Mn oxide production also demonstrated that the recombinant strain M15-pQE-*cotA* had greater Mn(II) oxidation activity than the mother strain M15.

Most importantly, a clear impression could be gotten from the SEM photographs that more Mn oxides were accumulated on the recombinant strain M15-pQE-*cotA* than the mother strain M15. Therefore, all these convincing proofs helped to verify our initial hypothesis that CotA from *B. pumilus* WH4 was responsible for the Mn(II) oxidation, which firstly established the immediate linkage between the bacterial MCOs and Mn(II) oxidation, raising a new and intriguing question about the fundamental role of other MCOs played in those different Mn(II)-oxidizing bacteria.

### What’s the Mechanisms of Bacterial Mn(II) Oxidation?

Until now, the structures and compositions of biogenic Mn oxides as well as the molecular mechanisms of bacterial Mn(II) oxidation, have remained largely a mystery. The available data only suggests that the oxidation of Mn(II) may involve a unique MCO system that contains three types of copper binding sites with different spectroscopic and functional properties [5]. The mononuclear Type 1 blue copper (T1) site is the primary electron acceptor from the substrate *via* two histidine and one cysteine residue (Figure S7C). Electrons are then transferred to a trinuclear cluster consisting of one type 2 (T2) and two type 3 copper (T3a/T3b), which serves as the oxygen binding and reduction site (Figure S7C), and from there four electrons (from four substrate molecules) ultimately reduce O_2_ to 2H_2_O [3], [6]. Nevertheless, in a chemical reaction, this process of MnO_2_ production requires a two-electron oxidation of Mn(II). Why are MCOs only known to engage one-electron transfers from substrate to O_2_? Bioinorganic chemists and microbiologists have long been interested in this process, and finally they have trapped the one-electron oxidation product-Mn(III) in experiments with the exosporium of a marine *Bacillus* sp. strain SG-1 [48], [49]. It has been demonstrated that enzymatic Mn(II) oxidation proceeds *via* two one-electron steps: 1) oxidation of Mn(II) to Mn(III) and 2) oxidation of Mn(III) to Mn(IV). Both oxidation steps are catalyzed by the same enzyme and the Mn(II) to Mn(III) step is the rate-limiting step. Mn(III), which occurs as a transient intermediate, can be captured and stabilized by the organic or inorganic ligands (L) such as pyrophosphate, siderophore, small endogenous molecules and polypeptides, to form a soluble Mn(III) complex (Mn(III)–L). This complex is either stable in solution or undergo oxidation or disproportionation to Mn(IV) oxides [3]. It is probable that the Mn(II) oxidation mechanism interpreted by CotA *in vitro*, will proceed along similar lines just like the study in *Bacillus* sp. strain SG-1, producing an enzyme-bound Mn(III) intermediate. However, the nature of the enzyme-Mn(III) intermediate and how the MCO catalyzes both oxidation steps, are still unknown. Thus, using purified enzymes to investigate this biochemically unique process will be much easier and helpful.

### CotA is an Appropriate Candidate for Biotechnological Applications and Needs Further Study

Although the present study has given the ultimate confirmation that the purified recombinant CotA protein possess the ability to oxidize Mn(II), more available evidences still need to verify its function and clarify the underlying Mn(II) oxidation mechanism. Whether it is the only Mn(II) oxidase in *B. pumilus* WH4? The answer is unknown, which demands us to put more efforts in the future research. Recently, two Mn(II) oxidases, McoA (belong to the bilirubin oxidase MCO superfamily) and MnxG, have been demonstrate to be necessary for Mn(II) oxidation in *P. putida* GB-1 [50]. In-frame deletions of either loci resulted in strains that retained some ability to oxidize Mn(II) or Mn(III), loss of oxidation was only attained upon deletion of both genes. If there are multiple Mn(II) oxidase enzymes in *B. pumilus* WH4 as well, it can be readily explained that why the deletion of *cotA* had no effect on the Mn (II)-oxidizing activity in *B. subtilis* [20], possibly because the independent two or more MCO enzymes dominate under different growth conditions and the loss of Mn(II) oxidation may be complemented by the residual Mn(II) oxidase. Therefore, in order to prove this hypothesis on the co-existence of MCOs, a lot of molecular manipulations should be carried out, involving the conventional gene knockout approach.

On the other hand, as a bacterial laccase, CotA might offer great potentials as biocatalysts in biotechnological and industrial applications, such as the decolorization of textile dyes and oxidation of a variety of organic and inorganic substrates. Additionally, CotA showed a markedly higher affinity for bilirubin than conventional bilirubin oxidase and catalyzed the oxidation of bilirubin to biliverdin [51], which could be used clinically to determine the levels of total and conjugated bilirubin in serum [51].

CotA could also play a particular role in the economically favorable removal of Mn(II) from groundwaters. Moreover, the biogenic Mn oxide particulates or spores that produced by CotA protein or *B. pumilus* WH_4_ could be acted as effective heavy metal adsorbents [38], [52]. The Cr(III) or Cd oxidation capacities of biogenic Mn oxides were 0.24 mmol g^−1^ and 2.04 mmol g^−1^, respectively, which even higher than the chemically synthesized Mn oxides in the aquatic environment [38], [52]. Such studies would be greatly helpful in the feasibility and designing of industrial-scale bioreactors for treating heavy metals contaminated wastewater, and highlight the potential for the application of this bioremediation friendly system, as products could be removed from effluents in the form of a precipitate [8].

## Supporting Information

Figure S1 **A diagrammatic representation of the operon structure for MCO genes from various strains.** Depicted as white arrows are the genes that encode the putative Mn(II) oxidase of *Bacillus* sp. strain SG-1 [11], *L. discophora* SS-1 [12], *Pedomicrobium* sp. ACM 3067 [14] and *B. pumilus* WH4 (this study). While neighboring genes are shown by grey arrows. Gene names, when available, are listed above the genes and the putative functions of the non-MCO genes are below. Cu^2+^ binding sites are marked with black rectangles and are lettered according to sequence homology.
(TIF)

Figure S2 **SDS-PAGE analysis of CotA expression and purification.** Lanes 1 and 2, whole cell protein fractions of M15-pQE-*cotA* induced without and with IPTG; lanes 3 and 4, the soluble extract and the precipitate after disruption with a French pressure cell; lane 5, the uncombined soluble extract after loading onto Ni-NTA agarose column; lane 6, the last effluent liquid with wash buffer (20 mM Tris-HCl (pH 8.0), 500 mM NaCl, and 80 mM imidazole); M, molecular size markers; lanes 7–11, first five tubes of CotA eluates (1 ml each); lane 12, the elution fractions were dialyzed against a buffer containing 50 mM Tris-HCl (pH 7.9) and 500 mM NaCl.
(TIF)

Figure S3 **The laccase activity assays of purified CotA by oxidizing three different substrates.** (A) The ABTS test was performed in 100 mM citrate-phosphate buffer (pH 4.0) with (tube 1) and without (tube 2) CotA. (B) The SGZ test was performed in 100 mM phosphate buffer (pH 6.0) with (tube 1) and without (tube 2) CotA. (C) The 2,6-DMP test was performed in 100 mM citrate-phosphate buffer (pH 5.0) with (tube 1) and without (tube 2) CotA.
(TIF)

Figure S4 **The optimal parameters for the oxidation of ABTS by CotA.** (A) The pH-dependent activity profile. The assay was determined at 37°C in 100 mM citrate-phosphate buffer (pH 3.0–8.0) supplemented with 0.5 mM ABTS and CotA. (B) Effect of temperature on the ABTS oxidizing activity. The optimum temperature was performed in 100 mM citrate-phosphate buffer (pH 4.0) supplemented with 0.5 mM ABTS and CotA at temperatures ranging from 30 to 100°C. (C) The optimal cooper concentration. The experiment was tested by adding CuCl_2_ (0–3 mM) to the 100 mM citrate-phosphate buffer (pH 4.0) supplemented with 0.5 mM ABTS and CotA at 37°C. The values were means ± standard deviations for triplicate assays.
(TIF)

Figure S5 **Mn(II) adsorption and oxidation on K plates by IPTG induced** ***E. coli*** **strains.** (A) The recombinant strain M15-pQE-*cotA* cultured with (plate 2) and without (plate 1) 5 mM Mn(II). (B) LBB test (plate 1–2) for the production of Mn oxides corresponds to plate 1–2 of panel A, respectively. (C) The mother strain M15 cultured with (plate 2) and without (plate 1) 5 mM Mn(II). (D) LBB test (plate 1–2) for the production of Mn oxides corresponds to plate 1–2 of panel C, respectively.
(TIF)

Figure S6 **Multiple amino acid sequence alignments of CotA proteins from** ***B. pumilus*** **WH4 (B.p.WH4),** ***B. pumilus*** **ATCC 7061 (B.p.ATCC7061),** ***B. subtilis*** **168 (B.s.168) and** ***B. licheniformis*** **ATCC 14580 (B.l.ATCC14580) using Clustal Omega software.** Highly conserved regions are boxed. Within those, invariant residues are represented against a red background. The copper-binding regions A, B, C and D are represented in blue color, and the conserved copper-binding residues are marked with asterisks (★).
(TIF)

Figure S7 **Three-dimensional structure model of CotA from** ***B. pumilus*** **WH4.** (A) The homology model of CotA. It is constructed using SWISS-MODEL program based on its homologous template CotA from *B. subtilis* (2WSD). α-helix (red), β-sheet (yellow), loop (blue) as well as 4 copper ions (cyan) are shown in the structure. (B) Residues which are involved in copper ion (cyan) binding (H103, H105, H151, H153, H419, H422, H424, H491, C492, H493, H497 and M502) are shown as gray sticks. (C) The coordination bonds among the 4 copper atoms and the 12 conserved amino acid residues (H103, H105, H151, H153, H419, H422, H424, H491, C492, H493, H497 and M502) of the CotA (see Figure 1) are shown in plane (the diagram was constructed by the method described in reference [39].
(TIF)
