# Supplementary figures and images for: CotA, a Multicopper Oxidase from Bacillus pumilus WH4, Exhibits Manganese-Oxidase Activity
Source: PLoS One. 2013 Apr 5;8(4):e60573. doi: 10.1371/journal.pone.0060573 (PMC3618234; doi:10.1371/journal.pone.0060573)

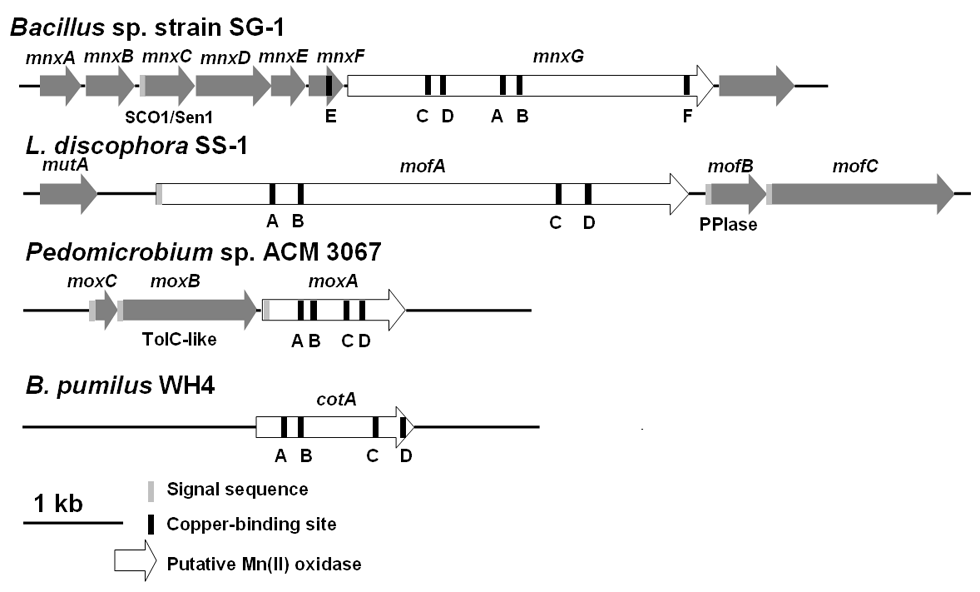

Supplement: Figure S1 — A diagrammatic representation of the operon structure for MCO genes from various strains. Depicted as white arrows are the genes that encode the putative Mn(II) oxidase of Bacillus sp. strain SG-1 [11], L. discophora SS-1 [12], Pedomicrobium sp. ACM 3067 [14] and B. pumilus WH4 (this study). While neighboring genes are shown by grey arrows. Gene names, when available, are listed above the genes and the putative functions of the non-MCO genes are below. Cu2+ binding sites are marked with black rectangles and are lettered according to sequence homology. (TIF) [file pone.0060573.s001.tif]

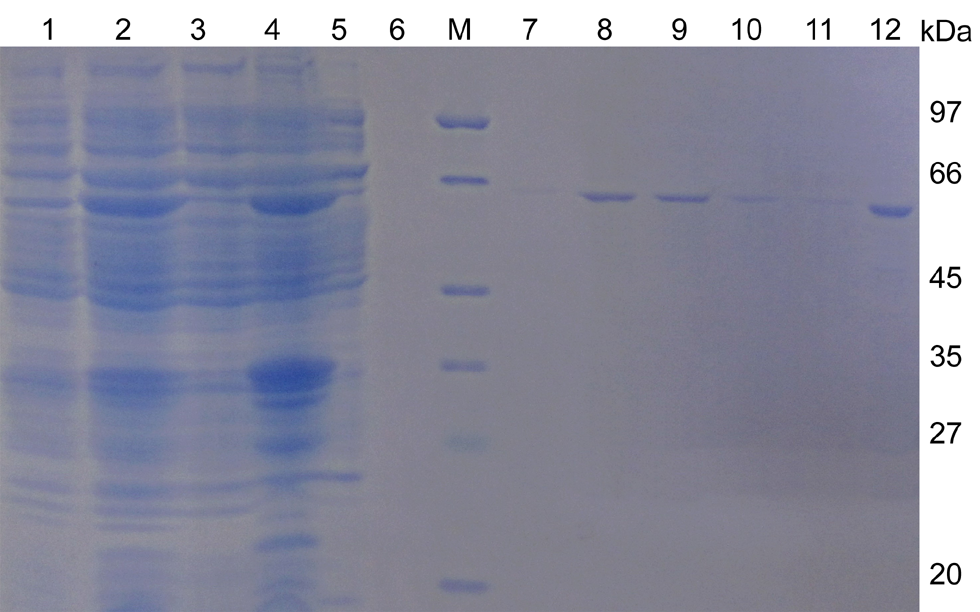

Supplement: Figure S2 — SDS-PAGE analysis of CotA expression and purification. Lanes 1 and 2, whole cell protein fractions of M15-pQE-cotA induced without and with IPTG; lanes 3 and 4, the soluble extract and the precipitate after disruption with a French pressure cell; lane 5, the uncombined soluble extract after loading onto Ni-NTA agarose column; lane 6, the last effluent liquid with wash buffer (20 mM Tris-HCl (pH 8.0), 500 mM NaCl, and 80 mM imidazole); M, molecular size markers; lanes 7–11, first five tubes of CotA eluates (1 ml each); lane 12, the elution fractions were dialyzed against a buffer containing 50 mM Tris-HCl (pH 7.9) and 500 mM NaCl. (TIF) [file pone.0060573.s002.tif]

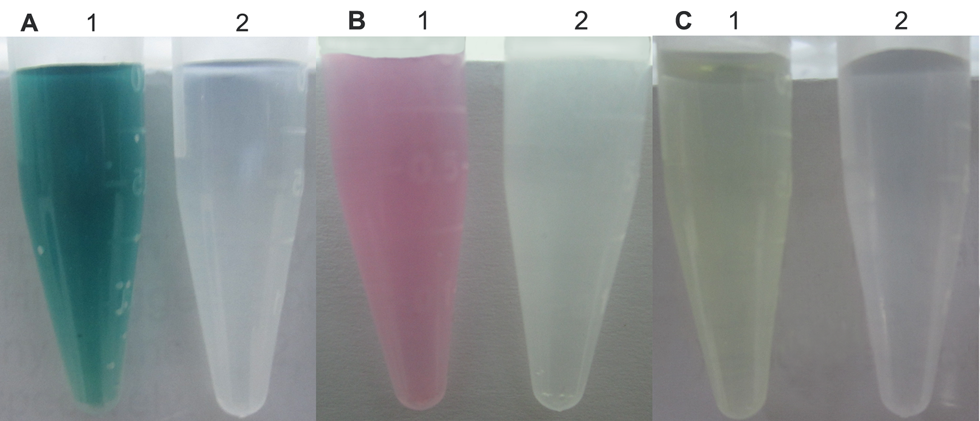

Supplement: Figure S3 — The laccase activity assays of purified CotA by oxidizing three different substrates. (A) The ABTS test was performed in 100 mM citrate-phosphate buffer (pH 4.0) with (tube 1) and without (tube 2) CotA. (B) The SGZ test was performed in 100 mM phosphate buffer (pH 6.0) with (tube 1) and without (tube 2) CotA. (C) The 2,6-DMP test was performed in 100 mM citrate-phosphate buffer (pH 5.0) with (tube 1) and without (tube 2) CotA. (TIF) [file pone.0060573.s003.tif]

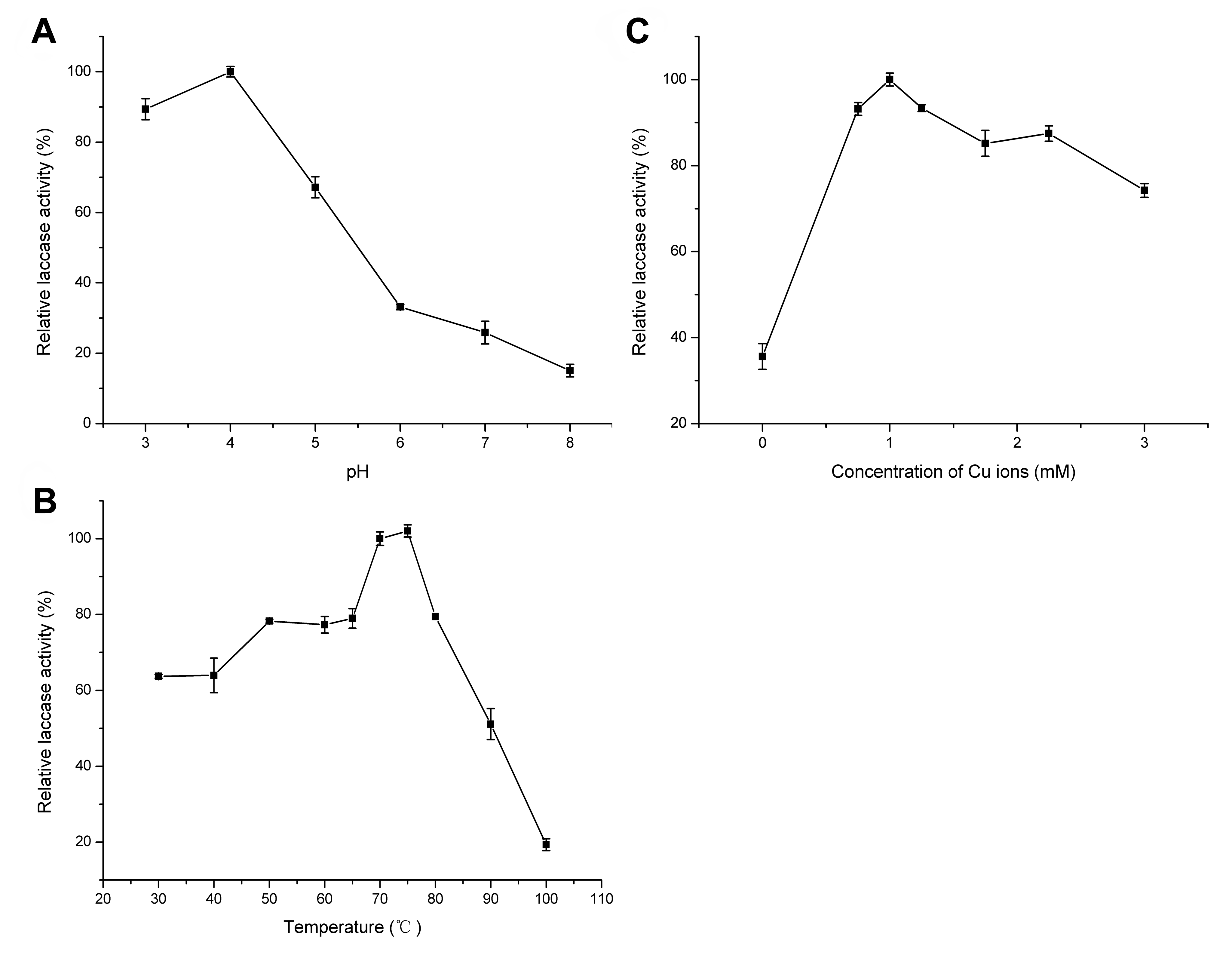

Supplement: Figure S4 — The optimal parameters for the oxidation of ABTS by CotA. (A) The pH-dependent activity profile. The assay was determined at 37°C in 100 mM citrate-phosphate buffer (pH 3.0–8.0) supplemented with 0.5 mM ABTS and CotA. (B) Effect of temperature on the ABTS oxidizing activity. The optimum temperature was performed in 100 mM citrate-phosphate buffer (pH 4.0) supplemented with 0.5 mM ABTS and CotA at temperatures ranging from 30 to 100°C. (C) The optimal cooper concentration. The experiment was tested by adding CuCl2 (0–3 mM) to the 100 mM citrate-phosphate buffer (pH 4.0) supplemented with 0.5 mM ABTS and CotA at 37°C. The values were means ± standard deviations for triplicate assays. (TIF) [file pone.0060573.s004.tif]

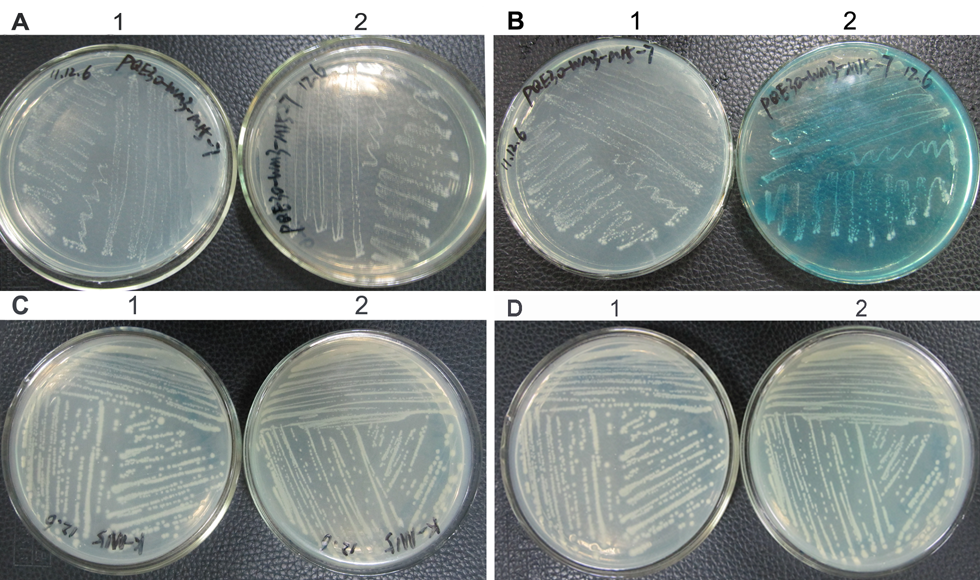

Supplement: Figure S5 — Mn(II) adsorption and oxidation on K plates by IPTG induced E. coli strains. (A) The recombinant strain M15-pQE-cotA cultured with (plate 2) and without (plate 1) 5 mM Mn(II). (B) LBB test (plate 1–2) for the production of Mn oxides corresponds to plate 1–2 of panel A, respectively. (C) The mother strain M15 cultured with (plate 2) and without (plate 1) 5 mM Mn(II). (D) LBB test (plate 1–2) for the production of Mn oxides corresponds to plate 1–2 of panel C, respectively. (TIF) [file pone.0060573.s005.tif]

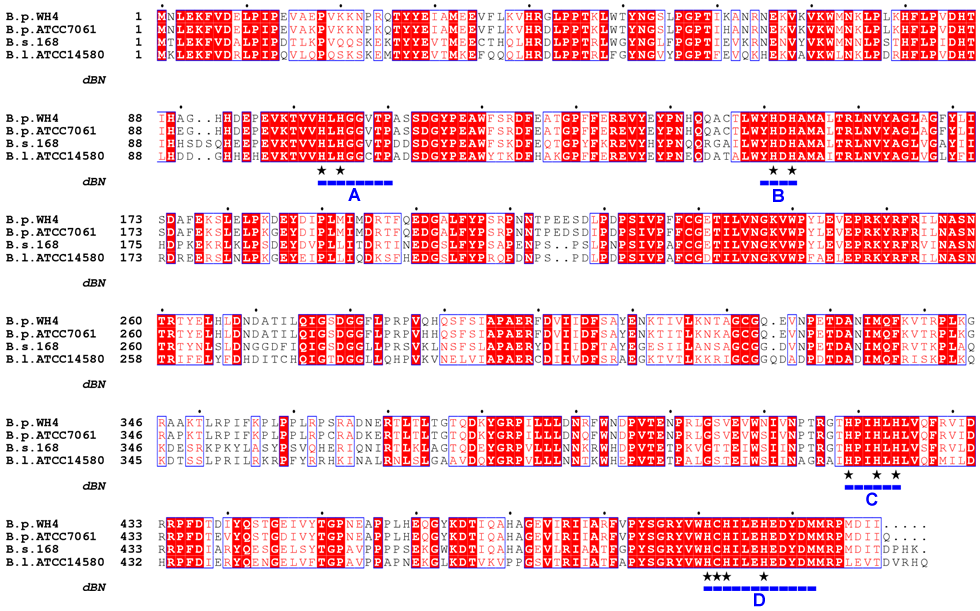

Supplement: Figure S6 — Multiple amino acid sequence alignments of CotA proteins from B. pumilus WH4 (B.p.WH4), B. pumilus ATCC 7061 (B.p.ATCC7061), B. subtilis 168 (B.s.168) and B. licheniformis ATCC 14580 (B.l.ATCC14580) using Clustal Omega software. Highly conserved regions are boxed. Within those, invariant residues are represented against a red background. The copper-binding regions A, B, C and D are represented in blue color, and the conserved copper-binding residues are marked with asterisks (★). (TIF) [file pone.0060573.s006.tif]

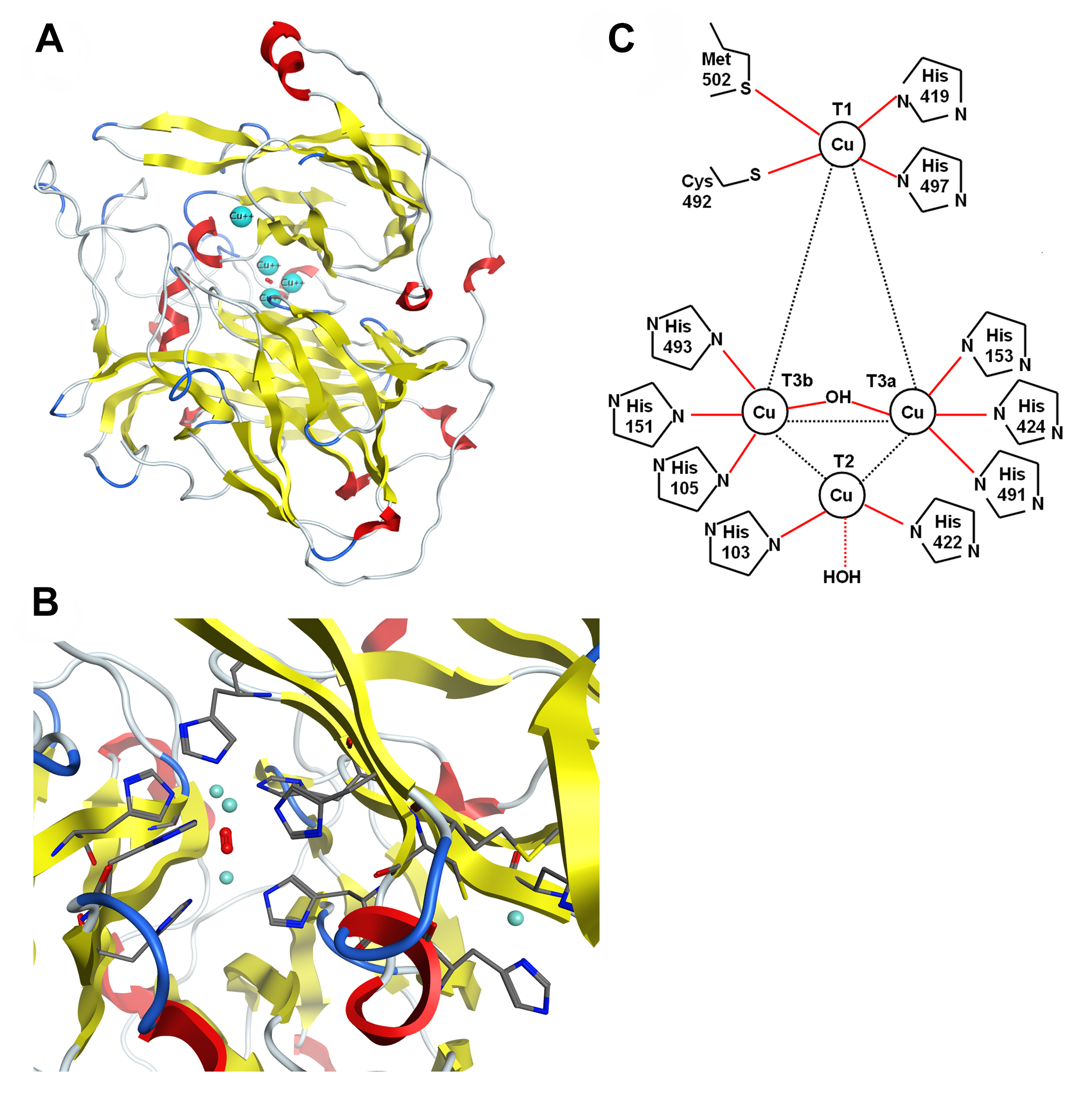

Supplement: Figure S7 — Three-dimensional structure model of CotA from B. pumilus WH4. (A) The homology model of CotA. It is constructed using SWISS-MODEL program based on its homologous template CotA from B. subtilis (2WSD). α-helix (red), β-sheet (yellow), loop (blue) as well as 4 copper ions (cyan) are shown in the structure. (B) Residues which are involved in copper ion (cyan) binding (H103, H105, H151, H153, H419, H422, H424, H491, C492, H493, H497 and M502) are shown as gray sticks. (C) The coordination bonds among the 4 copper atoms and the 12 conserved amino acid residues (H103, H105, H151, H153, H419, H422, H424, H491, C492, H493, H497 and M502) of the CotA (see Figure 1) are shown in plane (the diagram was constructed by the method described in reference [39]. (TIF) [file pone.0060573.s007.tif]
